# Positive correlation of cg16672562 methylation with obesity-related traits in childhood obesity, and its independence with underlying *HIF3A* (hypoxia-inducible factor 3a) genetic background

**DOI:** 10.18632/oncotarget.18707

**Published:** 2017-06-27

**Authors:** Suman Lee, Hyo Jin Kim, Sohee Han, Jae-Pil Jeon, Sang-Ick Park, Ho-Yeong Yu, Mi Yeong Hwang, Juyoung Lee

**Affiliations:** ^1^ Center for Genome Science, National Institute of Health, Chungju, Chungcheongbuk-do, 361-951, Republic of Korea; ^2^ Center for Biomedical Sciences, National Institute of Health, Chungju, Chungcheongbuk-do, 361-951, Republic of Korea

**Keywords:** cg16672562, *HIF3A*, obesity, CpG methylation, cis-meQTL

## Abstract

Differential methylations of the *HIF3A* (hypoxia-inducible factor 3a) gene have been linked to body mass index (BMI). To explore the association of these methylations to childhood obesity, we measured 5 CpG methylation sites (cg27146050, cg46801562, cg22891070, cg16672562 and cg46801675) in intron 1 of the *HIF3A* gene by pyrosequencing, in the Korean population (mean age: 13.9 yrs, 305 obese cases and 387 controls). Two CpG methylations, cg46801562 and cg16672562, had statistically significant association with childhood obesity (*P* = 2.09E-9 and 1.66E-7, respectively). Notably, in the case of cg16672562, all correlations were significantly positive with BMI (*beta* = 0.285, *P* = 1.652E-13), waist-hip ratio (*beta* = 0.0028, *P* = 1.42E-15) and fasting plasma glucose level (*beta* = 0.0645, *P* = 2.61E-4), when analyzed by linear regression, with age and sex as covariates. We investigated any genetic effect of cg16672562 methylation by using 14 single nucleotide polymorphisms (SNP) identified by exome sequencing of the HIF3A locus cg16672562 methylation showed no statistically significant changes due to the 14 polymorphisms. In this study, we show that cg16672562 is the most significant blood DNA methylation marker for childhood obesity in the Korean population, and might be independent of any underlying *HIF3A* genetic background.

## INTRODUCTION

Most of the identified common genetic variants for complex diseases appear to confer a modest degree of risk, and few causal alleles have been identified [1]. Obesity is largely hereditary, but the genetic basis of obesity is difficult to fully explain. The effect size of the known genetic associations for obesity is small; thus, epigenetic influences on gene regulation may be an important factor in susceptibility to obesity [2]. Genetics, epigenetics, and environmental exposure are likely to interact and contribute to an individual’s risk of obesity [3].

An epigenome-wide association study (EWAS) of obesity showed that differential methylation in intron 1 of the hypoxia-inducible factor 3a (*HIF3A*) gene in blood were associated with increased body mass index (BMI) [4]. Alterations of DNA methylation at three CpG sites (cg22891070, cg27146050 and cg16672562) in the DMR (differential methylation region) located in intron 1 of *HIF3A* were associated with BMI. Other EWAS study showed that significant hypermethylation of CpG sites (cg22891070 and cg46801699) at positions chr 19: 46801642 and 46801699 near a site in intron 1 of the *HIF3A* gene were associated with childhood obesity in a Chinese population and were correlated with alanine aminotransferase (ALT) levels [5]. There is a positive significant association between *HIF3A* methylation and BMI, but it is not clear whether differential methylation of *HIF3A* is a cause or consequence of obesity. Pan *et al*. found that *HIF3A* methylation measured in umbilical cord blood was associated with neonatal birth weight and adiposity, which suggests that the differential methylation of *HIF3A* is not solely a consequence of acquired adiposity [6]. Richmond *et al*. showed that childhood BMI was significantly associated with *HIF3A* methylation in adolescence in a longitudinal analysis of ∼1,000 mother-offspring pairs, which supports the notion that BMI affects *HIF3A* methylation [7].

Methylation of the differential methylation positions (DMPs) was dependent on sequential variations located near the DMPs. Two single nucleotide polymorphisms (SNPs), rs8102595 and rs3826795, were reported to be associated with methylation changes at the HIF3A locus [4, 6, 8]. Dick *et al*. reported that two SNPs (rs8102595 and rs3826795) were independently associated with methylation at cg22891070 [4]. Huang *et al*. showed that rs3826795, which is a *cis-*methylation quantitative trait locus (*cis*-meQTL) of cg22891070, was associated with changes in BMI through interactions with total or supplemental vitamin B2, vitamin B12, and folate [8].

Excessive accumulation of adipose tissue surrounding peripheral organs is a hallmark of obesity. This phenomenon can reduce oxygen pressure, leading to hypoxia and accompanying abnormal inflammatory and metabolic responses [9]. Obesity can cause the human body to enter a state of systemic hypoxia. Hypoxia-inducible factor (HIF), a master regulator of the adaptive response to hypoxia, is involved in the pathogenesis of obesity. *HIF3A* may function to accelerate adipogenesis [10]. *HIF3A* methylation and its expression in adipose tissues is fat deposit-specific and are related to obesity and adipose tissue dysfunction [11]. HIF pathways may play important roles in the development of adipose tissue dysfunction in obesity.

In this study, we investigated genetic and epigenetic variations of the *HIF3A* gene using exome sequencing and target pyrosequencing of up to 692 Korean childhood obesity subjects. We looked at obesity-related differentially methylated regions (five CpG sites: cg27146050, cg46801562, cg22891070, cg16672562, and cg46801675) by pyrosequencing to investigate their associations with glycemic and obesity related traits. Our study suggests that in additions to BMI, the epigenetic perturbations of *HIF3A* gene were also linked to fasting glucose levels and waist-hip ratio. We found that cg16672562 is most significantly correlated with BMI in childhood obesity. By exome sequencing data of the *HIF3A* gene in matched subjects, our work suggested that the differential DNA methylation, especially cg16672562, was independent of any underlying HIF3A genetic background.

## RESULTS

### General characteristics of obese children and controls

A summary of the general characteristics of obese children and controls is given in Table 1. The study subjects included extremely obese children (n = 305, average BMI of 31.71 ± 3.94) and lean controls (n = 387, average BMI of 19.44 ± 1.34) in the Korean Child-Adolescent Cohort Study (KoCAS). There was no significant difference between the two groups in age or gender (*P* > 0.05). The average waist-hip ratio (WHR) and fasting plasma glucose level (FPG) are also described, and, as Table 1 demonstrates, there are significant differences between cases and controls.

**Table 1:** Summary of the population characteristics for Korea childhood obesity

Variables	Controls	Cases	*P*
**Age**	13.93 ± 0.77	13.94 ± 0.84	0.6805
**N (M/F)**	387 (199/188)	305 (159/146)	0.8784
**BMI**	19.44 ± 1.34	31.71 ± 3.94	**<0.0001**
**WHR**	0.76 ± 0.05	0.89 ± 0.06	**<0.0001**
**FPG**	5.16 ± 0.39	5.23 ± 0.8	**<0.0001**

The values are indicated by mean ± standard deviation (SD), BMI: body mass index (kg/m^2^), FPG: fasting plasma glucose (mg/dL), WHR: waist-hip Ratio, *P* values <0.05 are described as bold characters.

**Figure 1:**
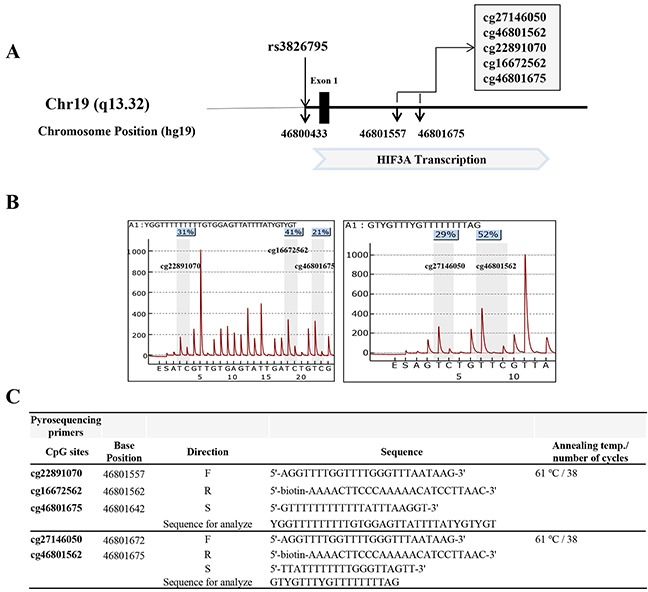
**(A)** The schematic diagram of rs3826795 and 5 CpG sites (cg27146050, cg46801562, cg22891070, cg16672562, and cg46801675) at the HIF3A gene. **(B)** The pyrograms of 5 CpG sites. **(C)** The primer sequences and PCR conditions for pyrosequencing.

We first investigated five DMPs in intron 1 (cg27146050, cg46801562, cg22891070, cg16672562, and cg46801675) of *HIF3A* by pyrosequencing. The genomic position and gene positions of the five target DMPs analyzed are described in Figure 1A. The five CpG sites in this study were located with 118 bp of each other (chr19:46801557 - chr19:46801675) and were all located in intron 1 of the *HIF3A* gene. rs3826795 (chr19:46800433) that previously known as association with cg22891070 was also noted [4]. Pyrograms for the five *HIF3A* target sites are shown in Figure 1B. The primer sequences and PCR conditions used are shown in Figure 1C. Finally, we examined five differential methylation positions (DMPs) in the control (n = 372) and obesity groups (n = 295). Details on the inclusion criteria are described in the Materials and Methods section.

### Analysis of target DMPs from controls and cases with childhood obesity

**Figure 2:**
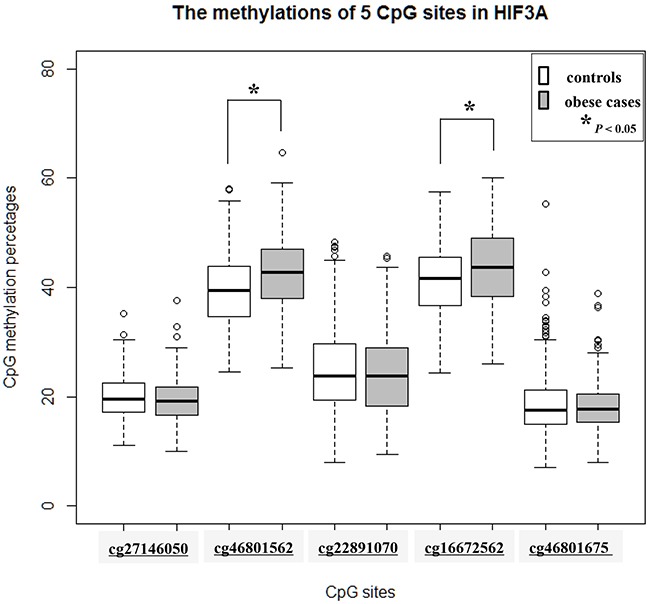
Boxplot of 5 CpG DNA methylation in Korean childhood obesity (n=667) CpG methylations between the control and cases were compared at 5 CpG sites (cg27146050, cg46801562, cg22891070, cg16672562, and cg46801675). Gray boxes indicate the obese cases. *P* values <0.05 is indicated as an asterisk (*).

The results of pyrosequencing analysis of all study subjects (n = 667, 372 controls and 295 obese cases) are given in Table 2. In particular, two CpG methylations, cg46801562 and cg16672562, were significantly associated with childhood obesity (*P* values: cg46801562 = 2.09E-9 and cg16672562 = 1.66E-7). The methylations between the controls and the obese cases of each CpG site were graphed at cg27146050, cg46801562, cg22891070, cg16672562, and cg46801675 sites in Figure 2. In particular, the degree of CpG methylation was increased by an average of 2.77 ∼ 3.1% at cg16672562 and cg46801562, whereas the other three sites (cg27146050, cg22891070, and cg46801675) exhibited relatively slight changes in methylation between the controls and the obese cases (Figure 2). Finally, two differential CpG methylation sites (cg16672562 and cg46801562) in whole blood cells were strongly associated with childhood obesity.

**Table 2:** The association of 5 CpG methylations in intron 1 of *HIF3A* gene with childhood obesity

CpG sites	BP	Methylation % ofcontrols (n= 372)	Methylation % ofcases (n=295)	*P*
**cg27146050**	46801557	19.87±4.02	19.37±4.09	0.1019
**cg46801562**	46801562	39.50±6.67	42.6±6.65	**2.092E-9**
**cg22891070**	46801642	24.93±7.32	24.18±7.30	0.1927
**cg16672562**	46801672	41.07±6.45	43.84±6.81	**1.663-07**
**cg46801675**	46801675	18.58±5.78	18.0±4.28	0.154

The values are indicated by mean ± SD, *P* values <0.05 are described as bold characters, BP: base positions, OR: odds ratio.

### Correlations of target DMPs with obesity-related traits

We found that two DMPs (cg16672562 and cg46801562) are significantly associated with childhood obesity. We investigated the relationship between five differentially methylated DNA sites and other traits to uncover their biological significance. We examined the relationship of the DMPs with fasting plasma glucose level (FPG), BMI and waist-hip ratio (WHR) (n = 667). We used age and sex as the covariates for linear regression analysis. The beta values and *P* values between each CpG methylation and three traits (BMI, FPG, and WHR) are shown in Table 3.

**Table 3:** The correlations of 5 CpG methylations of *HIF3A* gene with glycemic and obesity related traits

		cg27146050	cg46801562	cg22891070	cg16672562	cg46801675
**BMI**	***beta***	0.0543	−0.235	0.022	0.2848	−0.034
	***P***	***0.0223***	*0.059*	*0.1932*	***1.652E-13***	*0.1895*
**WHR**	***beta***	−0.001	0.002	−0.0003	0.0028	−0.0001
	***P***	*0.0529*	*0.085*	***3.944E-08***	***1.42E-15***	***0.0495***
**FPG**	***beta***	0.0543	0.009	0.0684	0.0645	−0.044
	***P***	***3.859E-4***	***4.355E-4***	***2.2E-4***	***2.61E-4***	***3.81E-4***

BMI: body mass index (kg/m^2^), FPG: fasting plasma glucose (mg/dL), WHR: waist-hip Ratio, *P* values <0.05 are described as bold characters. Age and sex were used as covariates for linear regression analysis.

By linear regression analysis, two of the five DMPs had statistically significant correlations with BMI and FPG. cg16672562 and cg27146050 were positively correlated with BMI (*beta* values= 0.2848 and 0.0543, respectively) and FPG (*beta* values = 0.0944 and 0.0524, respectively). For every 1% increase in cg16672562 DNA methylation, BMI increased by 0.2848 kg/m^2^ and FPG by 0.0645 g/dL. We used extremely obese cases with an average BMI of 31.71 kg/m^2^, so our beta value is high compared to that reported in other studies [4]. All five DMPs were significantly associated with fasting blood glucose levels, with cg16672562 having the strongest correlation (*beta* value = 0.0645). Three DMPs had significant correlations with WHR, and only cg16672562 had a positive correlation, with a *beta* value of 0.0028 (*P* = 1.42E-15). Specifically, all DMPs were significantly correlated with glucose-related traits by linear regression analysis. The epigenetic signature of the *HIF3A* gene may be a cause or a result of the prolonged high blood glucose levels seen in cases of obesity. We found two DMPs that had significant associations with childhood obesity, but only cg16672562 was significantly correlated with all three measures (BMI, WHR and FPG).

**Figure 3:**
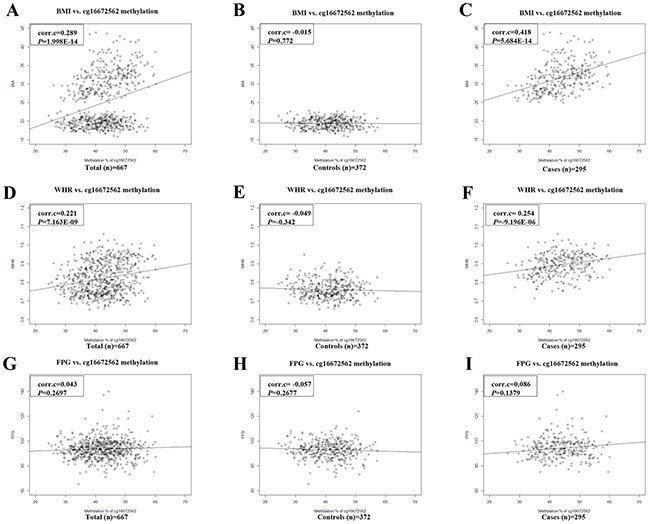
The correlation graphs of cg16672562 methylation with three traits in total, control and obese case groups **(A)** BMI in total, **(B)** BMI in controls, **(C)** BMI in obese cases, **(D)** WHR in total, **(E)** WHR in controls, **(F)** WHR in obese cases, **(G)** FPG in total, **(H)** FPG in controls, and **(I)** FPG in obese cases. Correlation coefficient (corr.c) and *P* value are indicated at each graph on top left.

The correlation of cg16672562 methylation with three traits was plotted for the total, control and obese case groups (Figure 3). The graphical correlation of cg16672562 with BMI is presented in total (Figure 3A), control (Figure 3B), and obese case group (Figure 3C). The cg16672562 methylation significantly correlated with BMI in the total and case group (correlation coefficient=0.289 and 0.481, respectively). The corr-elation of cg16672562 with WHR is shown in total (Figure 3D), control (Figure 3E), and obese case group (Figure 3F). The cg16672562 methylation significantly correlated with WHR in total and case group (correlation coefficient=0.221 and 0.254, respectively). In the control group, no significant correlation was observed with BMI and WHR (correlation coefficient= −0.015 and −0.049, respectively; *P* > 0.05). The correlations of cg16672562 with FPG in total (Figure 3G), control (Figure 3H), and obese case group (Figure 3I) are plotted. There was no correlation with FPG in any group, suggesting that age and sex may affect the correlation of FPG with cg16672562 methylation. By linear regression analysis with age and sex as covariates (Table 3), the correlation of cg16672562 with FPG was significant in the total group (beta=0.0645 and *P*=*2.61E-4*).

### Genetic association and *cis*-methylation quantitation loci (*cis*-meQTL) for 5 CpG sites

We performed exome sequencing of HIF3A locus on 692 blood samples. The method used is described in detail in the Methods section. We found a total of 14 sequencing variants from chr19: 46800000 to chr19: 46840000 (hg19) near cg16672562 positioned at chr19: 46801672; 5 SNPs were located in exons, 9 SNPs were in introns (Table 4).

**Table 4:** The associations of 14 SNPs with childhood obesity and their correlations with five CpG methylations of *HIF3A* gene

Genetic association with childhood obesity							The association of methylation quantitations with 14 SNPs									
							cg27146050		cg46801562		cg22891070		cg16672562		cg46801675	
SNP	BP	A1	GL	NO	OR	*P*	*beta*	*P*	*beta*	*P*	*beta*	*P*	*beta*	*P*	*beta*	*P*
rs3826795	46800433	G	intron	688	1.00	0.98	0.39	0.07	−0.63	0.09	0.77	0.05	0.62	0.10	0.42	0.14
rs4802306	46808371	T	intron	692	1.09	0.64	−0.59	**0.02**	0.21	0.64	−0.77	0.10	−0.46	0.31	−0.22	0.52
rs2311054	46811984	A	exon(syn)	692	0.90	0.60	0.63	**0.02**	−0.20	0.65	0.40	0.40	−0.24	0.60	−0.23	0.51
rs757638	46812126	A	intron	692	1.01	0.96	0.60	**0.02**	−0.13	0.77	0.32	0.50	−0.22	0.64	−0.21	0.54
rs759299	46812336	A	intron	692	0.95	0.79	−0.42	0.09	0.27	0.54	−0.20	0.66	0.42	0.33	0.43	0.19
rs61750957	46812451	T	exon(mis)	692	2.31	**0.01**	−0.35	0.46	0.45	0.58	1.14	0.19	0.97	0.24	0.75	0.23
rs79263603	46814003	G	intron	692	0.97	0.86	−0.06	0.79	0.20	0.63	0.13	0.78	−0.11	0.80	−0.20	0.53
rs8101216	46815297	T	intron	692	0.79	0.32	0.08	0.78	0.24	0.63	−0.41	0.45	−0.39	0.45	0.04	0.91
rs12461322	46815310	A	intron	692	1.00	0.98	−0.06	0.81	0.26	0.54	0.18	0.69	−0.05	0.91	−0.19	0.56
rs8101480	46815499	T	exon(syn)	692	0.83	0.43	0.07	0.82	0.30	0.54	−0.42	0.43	−0.36	0.48	0.05	0.90
rs3764609	46823702	G	exon(mis)	692	1.10	0.58	0.16	0.50	0.11	0.78	0.37	0.38	0.02	0.96	−0.29	0.34
rs3764610	46823751	T	exon(syn)	692	1.00	0.98	0.40	0.10	−0.21	0.60	0.15	0.74	−0.02	0.95	−0.20	0.53
rs77498043	46834320	A	intron	692	0.88	0.74	0.37	0.47	−1.04	0.24	−0.35	0.72	−0.27	0.77	−0.90	0.19
rs11665853	46834683	G	intron	692	1.10	0.58	0.17	0.48	−0.39	0.33	0.30	0.48	0.16	0.69	0.17	0.59

A1: minor allele, GL: gene Location, Sys: synonymous, mis: missense, BP: base positions, OR: odds ratio, NO: number of observations, *P* values <0.05 are described as bold characters. Test was done by additive effects of allele dosage. Age, sex and BMI were used as covariates for logistic and linear regression analysis.

By a genetic association study of 692 subjects (387 controls and 305 cases) with childhood obesity, and we found that one (rs61750957) of the fourteen SNPs was associated with childhood obesity (Table 4). rs61750957 was located in *HIF3A* exon 6, and associated with childhood obesity with odd ratio as 2.31 (*P* = 0.01). rs61750957 (Chr19: 46812451) is a missense polymorphic variation (Ala202Val), and also noncoding (nc) transcription variant (NC_000019.10:g.46309194C>T).

To investigate any genetic effects with 5 CpG methylations, we further performed *cis*-meQTL analysis by combining data of 14 SNPs with 5 CpG methylations. We found that only cg27146050 had statistically significant correlations with three SNPs, such as rs4802306, rs2311054 and rs757638 (Table 4). In the case of cg16672562, the methylation change was independent on the genotypes of 14 loci (Table 4). Dick *et al*. previously showed that rs3826795 and rs8102595, which are upstream of the DMR, are associated with methylation at cg22891070 [4]. Our exome sequencing data only included information on rs3826795, which show the marginal significance with cg22891070 methylation (*P*=0.05).

## DISCUSSION

We primarily focused on DMPs of the *HIF3A* gene that were previously reported in an Infinium study to be involved in adult obesity. Previous EWAS studies of BMI-related DMPs at the the HIF3A locus showed that increased methylation at the 3 CpG sites (cg22891070, cg27146050, and cg16672562) located is associated with increases or changes in BMI [4, 5, 7, 12]. The DMP in this region has already been reported to be involved in adult and childhood obesity, but a different method of quantification of CpG methylation was used (the Illumina 450K Bead Chip array [4, 12] versus the Sequenom MassARRAY system [5]. Wang *et al*. found cg22891070 (chr 19: 46801642) at the 3 CpG sites (cg22891070, cg27146050, and cg16672562) in the *HIF3A* gene (*P* < 0.05) in a study on Chinese childhood obesity. We identified an association between cg16672562 and extreme childhood obesity by pyrosequencing. The degree of methylation of cg16672562 is not comparable with that calculated using MassARRAY data of Wang *et al*. because the method did not determine changes in cg16672562 only [5]. In case of cg22891070, the difference in methylation between lean and obese children was found to be 24.18% and 24.93% by pyrosequencing (n = 667), and Sequenom’s MassARRAY data showed differences of 25.4% and 27.7% (n = 220) [5]. The BMI-related DMPs that we found are not present in all ethnic groups [12]. In particular, the effect of this region and its statistical significance are greater when the sample population consists of young high-BMI subjects.

Our data support previous reports on the epigenetic association of differential methylation in *HIF3A* in adults that illustrate the importance of differential DNA methylation of the *HIF3A* gene in obesity through changes in BMI. Methylation status of five CpG sites showed the significant associations with fasting plasma glucose level only after additional adjustment for age and sex. Overall, it suggests that these variables have influences on the investigated correlations of five CpG methylations with fasting plasma glucose level. Our findings may help improve the understanding of the contributions of epigenetic variation to the predisposition to diabetes.

Dick *et al*. reported that two SNPs (rs8102595 and rs3826795) were independently associated with methylation at cg22891070 [4]. Our data showed that rs3826795 has a marginal association with cg22891070 methylation (*P*=0.05), and other 13 SNPs neither have significant association (*P*>0.05). In general, our data showed that genetic variations located close to the DMR were not significantly associated with BMI-related DNA methylation changes, except cg27146050. A genetic context of the HIF3A locus might not be important for epigenetic modification of cg16672562 in high-BMI children. Further analysis of the newly-identified SNPs may be necessary to explain where and how the sophisticated DNA methylation changes come from, such as long range interactions [12].

## MATERIALS AND METHODS

### Study subjects

The study subjects, who ranged in age from 12 to 15 years, were selected from the KoCAS, which followed a cohort of Korean students using annual questionnaires and investigations at four elementary schools in Gwacheon from the time of the students’ entry into the school (IRB approval number, 2014-08EXP-05-P-A). The overall objective of the cohort study was to identify early risk factors for obesity and associated metabolic diseases in Korean children[13]. We defined “extreme obesity” as a BMI of ≥ 1.2 times the 95^th^ percentile value for the relevant age group or a BMI of ≥ 30 kg/m^2^, according to the recently proposed definition by the Centers for Disease Control and Prevention. Detailed characterizations of the study subjects are described in Table 1. Informed consent was obtained from the children’s parents.

### Pyrosequencing

Two independent PCR studies on bisulfite DNA were performed to identify five CpG sites (cg27146050, cg46801562, cg22891070, cg16672562, and cg46801675). cg46801562 and cg46801675 are named for their chromosome base pair locations (hg19). Target pyrosequencing of CpG sites combines a simple reaction protocol with a reproducible and accurate measure of the degree of methylation [13, 14]. Pyrosequencing assays were designed, optimized, and performed on the PSQ HS 96A System according to the manufacturer’s specifications (Qiagen, USA).

### Exome sequencing

All exons were captured by the SureSelect Human Exon V4 system (Agilent Technologies, Carlsbad, CA, USA) according to the manufacturer’s protocols. The captured DNA was sequenced with an Illumina HiSeq 2500. 100 bp paired-end exome sequencing was performed using a Genome Analyzer (Illumina Inc., San Diego, CA, USA). Base calling was conducted by the Illumina pipeline with the default parameter settings. All sequence data were assembled using the UCSC Genome Browser (GRCh37/hg19) and mapped by BWA (http://bio-bwa.sourceforge.net). Variant detection was performed using SAMTOOLS (http://samtools.sourceforge.net). The average depth of coverage was ∼67.2 fold.

### Statistics

To study DNA methylation replication using pyrosequencing, age sex, and BMI were used as covariates. Correlation coefficients between DNA methylation and other traits were calculated with Pearson’s tests and graphed. R scripts were used for all other analytical and graphical processing (http://www.r-project.org/). The genome-wide association study was analyzed using PLINK software (http://pngu.mgh.harvard.edu/∼purcell/plink).

## Abbreviations

EWAS: epigenome-wide association study
*HIF3A*: hypoxia-inducible factor 3a
BMI: body mass index (kg/m^2^)
DMPs: differential methylation positions
SNPs: single nucleotide polymorphisms
*cis*-meQTL: *cis-*methylation quantitative trait locus
FPG: fasting plasma glucose level (mg/dL)
